# Challenges for Nontechnical Implementation of Digital Proximity Tracing During the COVID-19 Pandemic: Media Analysis of the SwissCovid App

**DOI:** 10.2196/25345

**Published:** 2021-02-26

**Authors:** Viktor von Wyl

**Affiliations:** 1 Epidemiology, Biostatistics & Prevention Institute University of Zurich Zürich Switzerland; 2 Institute for Implementation Science in Health Care University of Zurich Zürich Switzerland

**Keywords:** epidemiology, normalization process theory, implementation, digital health, digital proximity tracing, digital contact tracing, COVID-19, app, surveillance, implementation, tracking, tracing, framework

## Abstract

**Background:**

Several countries have released digital proximity tracing (DPT) apps to complement manual contact tracing for combatting the SARS-CoV-2 pandemic. DPT aims to notify app users about proximity exposures to persons infected with SARS-CoV-2 so that they can self-quarantine. The success of DPT apps depends on user acceptance and the embedding of DPT into the pandemic mitigation strategy.

**Objective:**

By searching for media articles published during the first 3 months after DPT launch, the implementation of DPT in Switzerland was evaluated to inform similar undertakings in other countries. The second aim of the study was to create a link between reported DPT implementation challenges and normalization process theory for planning and optimizing complex digital health interventions, which can provide useful guidance for decision-making in DPT design and implementation.

**Methods:**

A Swiss media database was searched for articles on the Swiss DPT app (SwissCovid) published in German or French between July 4 and October 3, 2020. In a structured process, topics were extracted and clustered manually from articles that were deemed pertinent. Extracted topics were mapped to four NPT constructs, which reflected the flow of intervention development from planning, stakeholder onboarding, and execution to critical appraisal. Coherence constructs describe sense-making by stakeholders, cognitive participation constructs reflect participants’ efforts to create engagement with the intervention, collective actions refer to intervention execution and joint stakeholder efforts to make the intervention work, and reflexive monitoring refers to collective risk-benefit appraisals to create improvements.

**Results:**

Out of 94 articles deemed pertinent and selected for closer inspection, 38 provided unique information on implementation challenges. Five challenge areas were identified: communication challenges, challenges for DPT to interface with other processes, fear of resource competition with established pandemic mitigation measures, unclear DPT effectiveness, and obstacles to greater user coverage and compliance. Specifically, several articles mentioned unclear DPT benefits to affect commitment and to raise fears among different health system actors regarding resource competition. Moreover, media reports indicated process interface challenges such as delays or unclear responsibilities in the notification cascade, as well as misunderstandings and unmet communication needs from health system actors. Finally, reports suggested misaligned incentives, not only for app usage by the public but also for process engagement by other actors in the app notification cascade. NPT provided a well-fitting framework to contextualize the different DPT implementation challenges and to highlight improvement strategies, namely a better alignment of stakeholder incentives, or stakeholder-specific communication to address their concerns about DPT.

**Conclusions:**

Early experiences from one of the first adopters of DPT indicate that nontechnical implementation challenges may affect the effectiveness of DPT. The NPT analysis provides a novel perspective on DPT implementation and stresses the need for stakeholder inclusion in development and operationalization.

## Introduction

### The Use of Digital Tools for Pandemic Mitigation

The current SARS-CoV-2 pandemic is one of the first global events in which digital tools have played a prominent role in epidemiological crisis management [1,2]. Earlier attempts to operationalize digital tools for infection control included crowdsourced surveillance applications for influenza (eg, [3]) or the Zika virus [4]; however, these attempts often met with limited success due to recruitment or reporting adherence challenges [5-7]. In the current SARS-CoV-2 pandemic, digital tools that rely on passive contact sensing have gained significant traction to support manual contact tracing (MCT). Often referred to as digital proximity tracing (DPT), these technologies aim to facilitate contact tracing by storing proximity contacts or visited locations through apps or wristbands [8]. Some applications rely on a GPS to track movements; however, they have the downside of not preserving privacy (in cases in which the movement data are stored centrally) and only operate within the technical limitations of GPS (eg, accuracy limitations, limited operability in buildings) [9].

A more novel approach to proximity tracing is based on peer-to-peer tracking, such as through Bluetooth low energy beacons [10]. In this approach, apps send out signals that include a user-specific identification number, which are then received by smartphones within a certain radius [11]. The signal strength correlates with proximity (the stronger the signal, the closer the sending device), which can be leveraged to determine proximity contacts that occurred within a distance that potentially enables SARS-CoV-2 transmission. If one of the proximity contacts tests positive for SARS-CoV-2, all other app users with relevant proximity within the window of infectivity are warned by the app. While the accuracy is not perfect, it appears to be fit for the purpose of DPT [11,12], as also evidenced by reports of alerted app users who tested positive (eg, [13]).

### Implementation Challenges of DPT

Several countries are developing or employing DPT smartphone apps (eg, those listed in [9,14]). Early on, it was recognized that DPT needs to respect privacy [15], gain public trust [16], and adhere to ethical standards [17] to obtain a critical mass of app users required to have a significant impact on SARS-CoV-2 transmission [10]. Indeed, public surveys, some of which were conducted prior to app release, found that user acceptance of DPT strongly depends on privacy guarantees and public trust in app developers and sponsors [18-20]. Moreover, analyses of early DPT implementations, for example in Australia, suggest that technical aspects (eg, battery consumption, the need for apps to run in the foreground [21]) as well as persistent misconceptions [22,23] also play roles in user decisions and may hinder wide adoption. Therefore, privacy and trust deliberations have steered many of the academic [24] and public [25] debates as well as design decisions [26,27] related to DPT.

The following paragraphs of the introduction summarize some of these considerations before moving on to addressing the aims and contribution of this analysis. The first aim of this study was to compile and contextualize media debates regarding DPT implementation and effectiveness in Switzerland so that other countries can benefit from the experiences of one of the earliest DPT adopters. The second aim was to create a link between reported DPT implementation challenges with frameworks for analyzing complex digital health interventions, which can be useful to guide DPT design and (nontechnical) implementation decisions.

### Centralized Versus Decentralized DPT

In the beginning, two initiatives presented possible DPT architecture designs, and they differed by their degree of data decentralization. Decentralization means that Bluetooth-measured proximity contacts are only stored locally on the smartphone (an approach promoted and developed by the Decentralized Privacy-Preserving Proximity Tracing [DP-3T] consortium) [27]. By contrast, the centralized DPT concept proposed by the Pan-European Privacy-Preserving Proximity Tracing (PEPP-PT) initiative [26] also foresees contact information storage (static personal identifier numbers) on a central server, thus potentially enabling the gleaning of information about a user’s contact network [14]. These plans for centralized contact data storage led to privacy concerns and ensuing fears of lower population adoption rates, not only in public and academic circles [15] but also among major tech companies such as Google and Apple, who eventually decided to support the decentralized DPT architecture through the provision of smartphone application programming interfaces (APIs) [28]. As a consequence, many countries followed suit, with a majority of deployed DPT apps now being based on the decentralized DPT design [9]. In decentralized DPT, proximity contacts are evaluated locally on smartphones. The only data sent to a central server are anonymous random identifiers of persons with confirmed SARS-CoV-2 infection. These “infectious” identifiers are downloaded by all other users and compared against the locally stored identifiers of proximity encounters. If a match is found, users are notified and information on further steps is provided.

### DPT Is a Complement to Manual Contact Tracing

Manual contact tracing (MCT) is a cornerstone of many countries’ public health responses to SARS-CoV-2 (including Switzerland [29]). DPT is designed as a complement to MCT because it has distinct advantages. Specifically, DPT can warn exposed contacts much faster, warn multiple contacts simultaneously, and reach contacts not personally known to the index case [30]. By contrast, MCT identifies index case contacts through interviews, which is a labor-intensive process [31]. Given that exposed contacts enter quarantine upon app notification, the speed advantage of DPT should also lead to a faster interruption of transmission chains [10,32]. However, to achieve the desired goals, DPT needs adequate embedding in a country's overall test-trace-isolate-quarantine (TTIQ) response against the epidemic [29,33]. Furthermore, MCT remains indispensable because it enables better identification of transmission chains (eg, by including persons who do not use a DPT app) to assert prevention adherence (eg, compliance with mandatory isolation or quarantine) and obtain vital information about the time and setting of transmissions, thus providing valuable epidemiological data [31].

### Implementation of DPT in Switzerland

**Figure 1:**
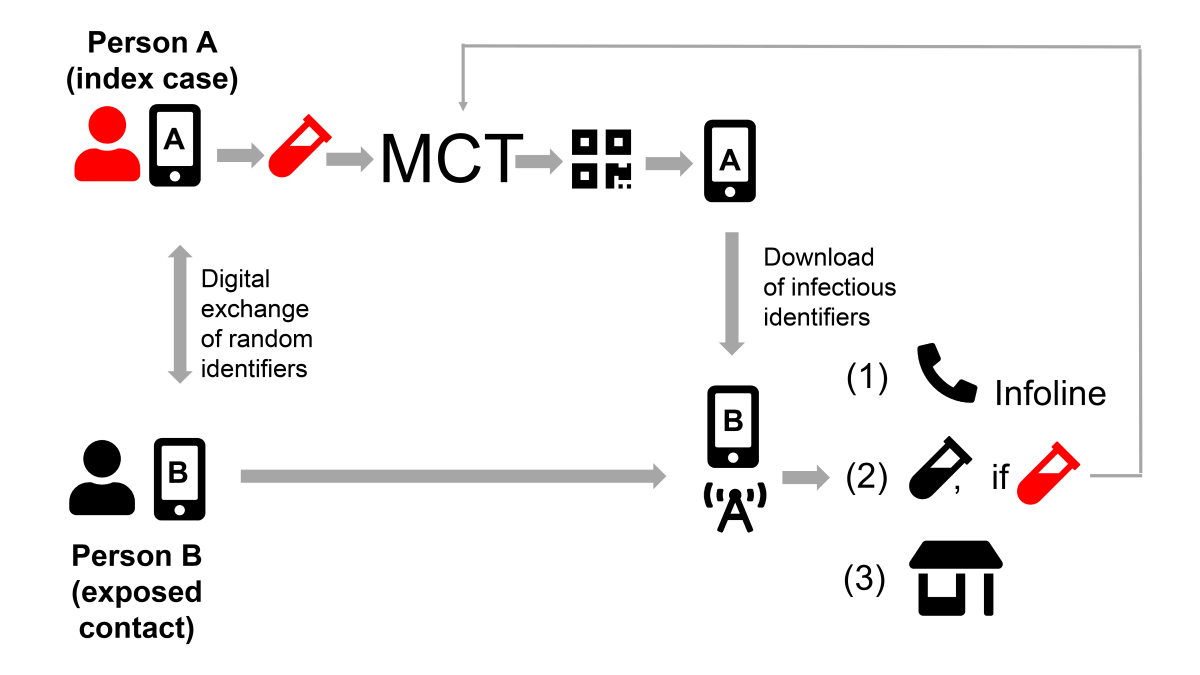
Steps in the notification cascade of digital proximity tracing. An infected person A tests positive for SARS-CoV-2 (red test tube), is referred to MCT, and receives and uploads a CovidCode to warn other app users. Person B was in close proximity and may have been infected. This person receives the app notification, upon which she has several options: calling an infoline (1), which is the recommended option, receiving a free test (2), and staying home voluntarily (3). MCT: manual contact tracing.

Switzerland was one of the first countries to release its own DPT app (the SwissCovid app) on June 25, 2020, based on the decentralized, privacy-preserving proximity tracing architecture (DP-3T) [27]. The overall principle of the SwissCovid notification cascade is illustrated in Figure 1. Of note, the app notification sequence involves multiple actors, including the testing laboratory, the physician ordering the test, MCT (which also generates the “CovidCodes,” that is, the authentication codes to be uploaded by the positive tested user), and an infoline that notified users are recommended to call. Although the SwissCovid infoline is centralized, MCT is organized at the level of the 26 cantons. The operational lead of MCT resides with the cantonal physician. Testing laboratories are decentrally organized private or public institutions. Guidelines and reporting forms are in place to inform the Federal Office of Public Health and the responsible cantonal health authorities about each newly detected SARS-CoV-2 case.

App-notified users have several options on how to proceed. The app notification suggests calling an official infoline. The infoline informs users that they are eligible for a free test after exposure notification and inquires more specifically about the potential risk of exposure. If indicated, the infoline recommends a voluntary quarantine (Step 1 in Figure 1). By comparison, MCT can result in order and enforcement of mandatory quarantine, but with salary compensation for working persons. Furthermore, notified app users can also directly request a free polymerase chain reaction (PCR) test (Step 2, Figure 1) or voluntarily stay at home without calling the infoline (Step 3, Figure 1), in which case they would not appear in any statistics. In the first phase after the app launch, the infoline could not directly refer callers to the responsible cantonal authorities and MCT; however, since September 2020, a respective agreement has been in place. Early on, the infoline was also confronted with erroneous calls; for example, some calls were triggered by an inaccurate translation of “weekly update notification” in Apple iOS 13.6 [34].

Some of the notification cascade steps have relatively strict timelines. Laboratories and attending physicians must report positive PCR test results to the responsible cantonal physician within two hours. The cantonal physician should generate the CovidCode and provide it to the infected app user, who has 24 hours to enter the code (after which the code expires).

### Known Challenges to the Implementation of DPT

In implementation science, complex interventions are defined as “consisting of multiple behavioral, technological, and organizational components” [35]. As illustrated in Figure 1, DPT fulfills this definition of complex public health interventions because it involves several steps from laboratory testing to communication of results, notification triggering, notification receipt, and quarantine. Consequently, the success of DPT hinges on an efficient cascade of notifications from positive PCR test results to proximity contacts and involves app users, SARS-CoV-2 testing laboratories, health authorities, and possibly other actors. Therefore, seamless integration of DPT into broader pandemic mitigation measures (eg, testing facilities) and high app user compliance with recommended measures (eg, trigger notifications, entering quarantine) is crucial, especially in settings with voluntary DPT usage.

Emerging data provide early evidence for the effectiveness and impact of DPT on the transmission dynamics of the pandemic [36,37]. For Switzerland, the first studies about the performance of SwissCovid, both in terms of individual notification cascade steps and overall, paint a mixed picture. A recent report demonstrated proof-of-principle for the technical infrastructure of DPT [13]. Specifically, the study reports on at least 60 persons who were tested after an app notification and who were found to test positive by PCR for SARS-CoV-2. However, the same report also highlights some inefficiencies in the notification chain as described above. For example, the number of CovidCodes exceeds the number of entered codes by approximately 50%, which—in part—is a result of the voluntary nature of DPT: at each step, users can select whether or not to use the app and undertake the recommended steps, without fear of retribution. Along similar lines, a separate report examined the uptake and reasons for nonuse of the SwissCovid app [23]. For example, higher monthly household income or being a nonsmoker were associated with higher SwissCovid app uptake, whereas older age, lack of trust in health authorities, or having a non-Swiss nationality correlated with a lower uptake. Furthermore, early media coverage of SwissCovid [25], as well as a systematic review [38], unearthed some organizational challenges for DPT implementation. This is not completely surprising given the novelty and complexity of the intervention. Combined, these reports underscore the relevance of nontechnical implementation aspects for optimal DPT functioning.

### Normalization Process Theory to Guide Planning and Implementation of Complex Digital Interventions

In other instances of complex intervention assessments, normalization process theory (NPT) has proven useful to systematically investigate the embedding of complex (digital) health interventions [35,39]. NPT aims to explain and promote factors that normalize an intervention, that is, to make it part of routine practice. NPT is centered around four core constructs, which also reflect the flow of an intervention development from planning, stakeholder onboarding, and intervention execution to critical appraisal [40]. Along this “life course” of an intervention, the *coherence* constructs describe how individual participants make sense of the implementation, *cognitive participation* reflects the participants’ collective efforts to create commitment and engagement with the intervention, *collective action* refers to the execution of the intervention and describes the joint efforts of all actors to make it work, and *reflexive monitoring* refers to collective appraisals of risks and benefits and developing improvements by all actors [40].

### Aims of the Analysis

To summarize, although some challenges and bottlenecks in DPT implementation are already known, a systematic compilation and framing of these implementation challenges is largely lacking. Furthermore, it remains unexplored whether and how generic digital health implementation frameworks help to conceptualize these DPT challenges and inform possible optimizations. Therefore, this analysis aimed to systematically scrutinize media reports on SwissCovid for statements and examples reflecting challenges to optimal intervention functioning. A major goal of this analysis was to identify and report challenges in the nontechnical implementation and embedding of DPT. These early experiences may inform other countries that are considering or actively implementing DPT about possible challenges and optimization strategies.

Furthermore, by mapping the identified challenges to the NPT constructs, the analysis also creates a link to the rich NPT literature, which provides tools and frameworks to optimize the adoption and effectiveness of digital health interventions.

## Methods

### Media Analysis

The present analysis was informed by Swiss media reports. The Swissdox Essentials media database was searched from July 4 to October 3, 2020 [41]. This database covers all Swiss print media and the most important web-based portals. Only entries in German or French, languages that are spoken by 85% of Swiss inhabitants [42], were considered. Using the search phrase *SwissCovid* or (*'Swiss Covid'* AND *app*), the Swissdox database was searched for unique print and web-based articles reflecting independent journalistic investigations. That is, reprinted articles or articles that referred to other articles without adding information were excluded. Live ticker transcripts were also excluded from our search.

Eligible articles were manually prescreened to determine whether they reported on problems or inefficiencies of the SwissCovid app. This screening process was facilitated by the Swissdox database, which highlighted all sentences containing the prespecified search terms. In a subsequent, more detailed screening, the selected pertinent news articles were read completely, and relevant sections were highlighted. During this process, duplicate articles were removed (along with those that simply paraphrased an earlier article), and topics were manually extracted based on the following predefined topic list (informed by subject knowledge of the author): problems referring to (a lack of) communication, technical problems or confusion regarding the app, issues related to the effectiveness of the app, delays in receiving test results, delays in receiving CovidCodes, issues related to the infoline, lack of support from participants, and competition for strained resources by SwissCovid. The key issues regarding the SwissCovid app were summarized manually and grouped by the author. All news article files are available from the author upon request.

### Linking of Reported Challenges With Normalization Process Theory

Finally, the key challenges reported in the press articles were contextualized and interpreted using the NPT questions developed by Murray et al [39]. NPT was selected a priori as an assessment framework based on findings in the literature. The present study followed the guiding questions outlined by Murray et al [39]. and considered the following stakeholders involved in the DPT notification cascade in Switzerland (henceforth also called “participants” to remain compatible with NPT terminology): app users; SARS-CoV-2 testing laboratories; cantonal health authorities and cantonal physicians who perform MCT; the Federal Office of Public Health (FOPH), which is the product owner of the SwissCovid app; and the infoline, operated on behalf of the FOPH by a commercial telehealth company. Mapping of DPT challenges to the different NPT constructs was performed manually by the author.

## Results

### Findings From the Media Analysis

**Figure 2:**
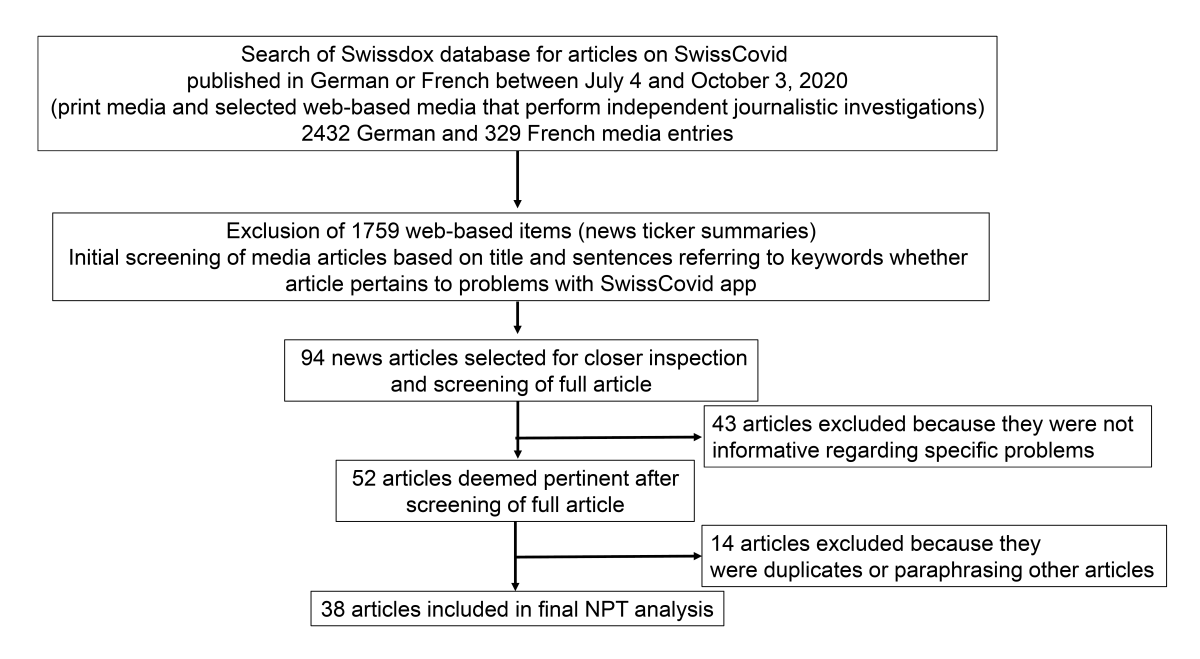
Flowchart of article selection from the Swissdox media database. NPT: normalization process theory.

Figure 2 outlines the search process of the media database, which resulted in a total of 38 articles deemed relevant for the analysis. The key topics extracted from the selected articles are outlined in Table 1 (with information on the 38 articles presented in Multimedia Appendix 1).

**Table 1:** Major topics identified in the selected articles.

Group	Name	Item number	Topic	Media reports^a^
A	Communication	A1	Need for optimization of app error messages	#22, #28, #53
		A2	Communication concerning DPT^b^ usage, privacy, and benefits could be improved	#10, #32
		A3	Unmet communication needs by some participants in the notification cascade, such as cantonal health authorities or physicians	#29, #14
B	Interfaces and processes	B1	Delays in CovidCode generation	#2, #8, #14, #17
		B2	Unclear connection between infoline and cantonal health authorities	#20
		B3	Legal hurdles for cantonal physicians to order a mandatory quarantine based on app notification	#29
		B4	Following a change of testing criteria, some confusion about procedures for obtaining free PCR^c^ test upon app notification	#14
		B5	Interference with work, such as nurses with proximity contacts to patients infected with SARS-CoV-2	#61, #35
C	Competition for resources	C1	Initial fears of cantonal authorities of being overwhelmed by persons receiving app notifications	#20
		C2	Concerns that app development and operation will drain resources from other pandemic mitigation efforts	#29, #30
D	Unclear effectiveness	D1	Some disappointment when download numbers started to plateau; fears that the number of active app users will not suffice for DPT effectiveness	#5, #18
		D2	Effect on pandemic mitigation still unclear for users and other participants, such as cantonal physicians	#18, #32, #14, #29
E	Obstacles to higher user coverage and compliance	E1	The app does not work abroad	#5, #22
		E2	Lingering fears regarding privacy	#11, #12, #25
		E3	Temporary lower infection numbers reduced the urgency for app usage	#12
		E4	PCR-positive app users are not entering the CovidCodes	#2, #28
		E5	App-notified users are not calling the infoline (and do not appear in any statistics	#57, #58
		E6	Lack of personal incentives to use or regularly check the app status (could be remedied, for example, by the inclusion of news updates about pandemic); The app remains silent unless there is a notification	#42, #48

^a^#ID refers to the individual media reports listed in Multimedia Appendix 1.

^b^DPT: digital proximity tracing.

^b^PCR: polymerase chain reaction.

### Topic Extraction

A more detailed analysis of the 38 pertinent articles revealed several challenges that largely fell into five topics: (1) communication challenges, (2) challenges to optimal DPT interfacing with other processes, (3) fear of competition for limited resources with established pandemic mitigation measures, (4) unclear effectiveness of DPT, and (5) obstacles to greater user coverage and compliance (Table 1).

Several articles cited communication challenges (group A, Table 1). Some reports referred to confusing app error messages, the need for intensified or improved communication to app users about the benefits and processes involved in SwissCovid, and a need for improved exchanges with other participants in the intervention, particularly the 26 cantonal health authorities. Most articles saw a solution for overcoming these challenges through intensified communication by the FOPH (being the SwissCovid product owner) with the public and other participants.

Regarding operational challenges (group B, Table 1), the most frequently echoed concern pertained to delays in sending CovidCodes to SwissCovid users with positive PCR tests for SARS-CoV-2. First media reports appeared in August 2020 after testimonies of persons who only received the codes after significant delays. Later articles also reported on procedural adjustments by cantonal health authorities to increase the speed of CovidCode generation and delivery.

In the context of delays of CovidCode generation, other processes, such as reporting of positive PCR test results by laboratories or access to testing, also came under scrutiny. Thereby, further potential problems surfaced that could affect the speed of the notification cascade. First, laboratories or physicians ordering PCR tests may sometimes be unable to adhere to the 2-hour timeline for communication of positive test results to cantonal authorities (eg, due to high testing volumes). Second, one physician stated that changing testing criteria and guidelines may have created some temporary confusion regarding procedures for accessing free testing by app-notified persons, which was resolved shortly after. Third, during the initial weeks after the app launch, the infoline was unable to refer app-notified callers directly to their respective cantonal health services for further evaluation. One article quoted a cantonal physician, who stated data protection reasons for this referral gap. Data protection was also stated as a reason why some health authorities found it difficult to integrate DPT into manual tracing procedures. According to one cantonal physician, the (intended) inability of DPT to provide additional data on the timing and place of potential exposure was diminishing its value for manual contact tracers.

Furthermore, some articles reported on challenges for health care workers to using the app, especially when they were engaged in the care of patients infected with SARS-CoV-2. Hospitals were concerned about frequent notifications (despite personnel wearing protective gear) and ensuing confusion. Some hospitals asked their employees to switch off SwissCovid while at work.

The third group of topics (group C, Table 1) concerned the resource situation on the part of the FOPH and the cantons. A retired FOPH official and at least two cantonal physicians were cited to have some doubts regarding DPT effectiveness and were therefore concerned that DPT would compete for scarce human resources at the FOPH and cantonal health authorities. The initial referral gap between the infoline and cantonal health authorities (cf group B) was, according to one source, driven by concerns of cantons of becoming overwhelmed by app-notified contacts.

The fourth cluster of challenges (group D, Table 1) pertained to a perceived unproven effectiveness of DPT. Because DPT was developed and released under immense time pressure and with limited real-world testing, doubts about the usefulness and contribution of DPT to pandemic mitigation persist. This uncertainty could potentially create a vicious cycle: the target population may not be inclined to use the app because of unproven effectiveness, but without widespread use, its effectiveness cannot be demonstrated. These concerns were echoed shortly after the public release of SwissCovid, when the number of active users seemed to plateau at approximately 1 million (on October 14, the SwissCovid app had 1.67 million active users and 2.5 million downloads [43]). This perceived lack of benefit was not confined to the public but also appeared to exist among some health authorities. Statements by two cantonal physicians alluded to views that DPT was considered to be an additional burden with unclear benefits by some health authority members.

The fifth cluster of topics (group E, Table 1) was related to user coverage and compliance. In July 2020, several media outlets reported on the plateauing (or even decreasing) user numbers as well as on discrepancies in the numbers of generated and uploaded CovidCodes (indicating that not all app users with positive PCR tests chose to trigger notifications). Several explanations were explored, such as lingering concerns about privacy (with a need for better communication), low overall case numbers of SARS-CoV-2 infections in July, or the inability to use the SwissCovid app abroad (eg, during vacation). A frequent conclusion by the media was a need for more communication by the FOPH to address these privacy concerns and to emphasize the potential benefits of the SwissCovid app. With increasing active SwissCovid use and the first manifestations of positive effects, these concerns moved somewhat to the background but never disappeared entirely.

### Mapping of Topics to NPT Constructs

Table 2 illustrates the mapping of identified topics to different NPT constructs. Overall, the media analysis provided information for most of the NPT questions. Except for topic E1 (“The app does not work abroad”), all topics mapped well to specific NPT constructs and individual subquestions. Of the individual topics, 10 fell into the *Coherence* construct, 5 into the *Cognitive Participation* construct, 10 into the *Collective Action* construct, and 3 into the *Reflexive Monitoring* construct.

**Table 2:** Mapping of topics to normalization process theory constructs.

Normalization process theory construct			Topic domains (cf Table 1)		Assessment
**Coherence (making sense of the intervention, ie, meaning and sense-making by participants)**					
	Is the intervention easy to describe?	D1, D2, E3, E6		DPT^a^ is difficult to explain; some misconceptions of what DPT should achieve (eg, generating helpful data) or requirements for success (eg, need of 60% participation rate in population to be successful).	
	Is it clearly distinct from other interventions?	C2		Because DPT is an adjunct to manual contact tracing, the distinction is not always obvious to all participants; DPT may even be seen as competition to manual tracing.	
	Does it have a clear purpose for all relevant participants?	C2, D2		There were doubts about the purpose of DPT or even the right to coexist with manual contact tracing.	
	Do participants have a shared sense of its purpose?	C2, D2		Not all participants are convinced, including parts of the population and cantonal health authorities.	
	What benefits will the intervention bring and to whom?	C1, D1, D2, E6		Benefits are abstract, not immediately visible, and partially context dependent (eg, role of second line of defense).	
	Are these benefits likely to be valued by potential participants?	D1, D2, E2, E4, E6		The overall potential benefits (slowing transmission) are valued by most, but doubts persist whether DPT can contribute toward that goal.	
	Will it fit with the overall goals and activity of [pandemic mitigation goals]^b^?	N/A^c^		DPT was designed to complement manual contact tracing; In principle, DPT is well aligned with other pandemic mitigation goals.	
**Cognitive participation (working out participation in the intervention, ie, commitment and engagement of participants)**					
	Are target user groups likely to think it is a good idea?	C1, C2, D1, D2		Some doubts seem to persist among all participants. Not all actors seem convinced of the benefits.	
	Will they see the point of the intervention easily?	E3		DPT was released during a time when case numbers were low. Benefits remained abstract and unclear, in part also because of low infection numbers. Initially, this may have affected the willingness to engage in DPT work processes.	
	Will they be prepared to invest time, energy, and work in it?	C1, C2		DPT was seen as competing for time and resources with other mitigation measures by some actors. Therefore, the willingness to engage in cognitive participation may have been limited.	
**Collective action (executing the intervention,** **ie, the work participants do to make the intervention function)**					
	How will the intervention affect the work of [participants]?	B1, B2, B3, B4, B5		DPT introduces additional steps and processes for MCT^d^. There were also some unclarities and frictions between different processes and interfaces (eg, between testing labs and cantonal physicians or between users with positive PCR^e^ tests and cantonal physicians).	
	Will it promote or impede their work?	B2, B5, C1, C2		DPT potentially adds to the workload of MCT; app use can be problematic for health care workers.	
	What effect will it have on [interactions]?	B2, B3, B4, C2		DPT notifications are an additional dimension to be covered in MCT interviews; interface between infoline and cantonal health authorities needed optimization.	
	Will staff require extensive training before they can use it?	A3, B4		In principle, yes, some reports indicate an additional need for instructions or communication for some (health system) actors.	
	How compatible is it with existing work practices?	C2, D2, E5, E6		DPT is seen as something separate that adds to the workload. Data protection apparently inhibits complete DPT integration into MCT. Notified users may take actions, but not always as recommended (eg, directly seeking tests).	
	What impact will it have on division of labor, resources, power, and responsibility between different professional groups?	B2, D2		Reports indicate several “interfacing” challenges, such as between infoline and cantonal health authorities. Reports of confusion regarding eligibility of free PCR testing for notified users.	
**Reflexive monitoring (reflecting on the intervention, ie, participants reflect on or appraise the intervention)**					
	How are users likely to perceive the intervention once it has been in use for a while?	D1, D2		Effectiveness still seems unclear or unproven for some actors. New case numbers of SARS-CoV-2 remained relatively low for most of the observation period, thus affecting perceived effectiveness.	
	Is it likely to be perceived as advantageous for [users and other participants]?	C2, D1, D2		This seems not to be the case for all actors.	
	Will it be clear what effects the intervention has had?	D1, D2		The uncertainty regarding DPT effectiveness hampers usage and implementation.	
	Can users/participants contribute feedback about the intervention once it is in use?	N/A, but indicated by some reports.		App users gave indirect feedback (eg, via social media). Other actors had direct interactions with the Federal Office of Public Health and the app developers.	
	Can the intervention be adapted or improved on the basis of experience?	N/A, but indicated by some reports.		App development is continuous; there is regular exchange about possible improvements.	

^a^DPT: digital proximity tracing.

^b^Expressions in brackets reflect adaptations of the original question wordings [39] to better fit the current analysis.

^c^N/A: not applicable.

^d^MCT: manual contact tracing.

^e^PCR: polymerase chain reaction.

The greatest concerns in the *coherence* construct pertained to unclear benefits and distinctions from other processes (especially manual contact tracing). These concerns were voiced by different participants, including app users and cantonal authorities. Additionally, fears of resource competition with other mitigation measures were cited. In part, these concerns reflected the complexity of the SwissCovid app notification cascade and possibly also an incomplete understanding of the role requirements by some participants.

Multiple reports also indicated challenges in the *cognitive participation* domain that overlap with other NPT constructs. Specifically, the interplay between different actors may require optimization, as well as clarifications regarding resource situations (eg, on the part of cantonal authorities).

Furthermore, many of the reported problems in the *collective action* domain indicated a need for optimized integration of SwissCovid into existing work processes. From the viewpoint of some cantonal health authorities, SwissCovid was perceived to add to the existing workload while providing unclear benefits. Additionally, some statements reflected a need for additional communication and knowledge transfers to different participants, for which the FOPH was seen to be in the lead.

By contrast, there were fewer media reports regarding the *Reflexive Monitoring* construct. However, some reports indicate that communication between different participants (FOPH, cantonal physicians, infoline operators) is ongoing, and optimizations have been planned or even put in place (eg, better coordination between infoline and cantonal health authorities; integration of news into the SwissCovid app to provide additional user incentives).

## Discussion

DPT is a complex public health intervention to mitigate the SARS-CoV-2 pandemic. Its effectiveness depends on appropriate embedding into a country's overall TTIQ strategy and requires multiple, timely actions by different actors. This article describes early experiences with DPT implementation in Switzerland, which has one of the longest track records of decentralized DPT operation.

Based on Swiss media articles published during the first three months after DPT release, this study presents different challenges in nontechnical DPT implementation. These challenges included unclear DPT benefits, which affected commitment and raised fears among different health system actors for resource competition with established pandemic mitigation measures. Moreover, media reports indicated process interface challenges in the notification cascade (eg, in the hand-over of app notified users from the infoline to responsible cantonal authorities), as well as misunderstandings and unmet communication needs on the side of some health system actors. Finally, some reports suggested misaligned incentives, both for app usage by the public as well as for process engagement by other actors in the app notification cascade [44].

### Challenges Viewed Through the Lens of NPT

In the SARS-CoV-2 pandemic, timely diagnosis, isolation, and quarantine are essential processes, which DPT apps are intended to support. However, procedural frictions can lead to delays, which also affect the effectiveness of DPT. Examples are late deliveries of CovidCodes (codes to trigger notifications) or uncertainties regarding access to PCR tests upon app notification. Furthermore, most challenges identified in the media search bear close relationships to the constructs of NPT. Specifically, many identified challenges mapped well to the *coherence* (“making sense of the intervention”) and *collective action* (“executing the intervention”) domains. For example, unmet communication and training needs regarding DPT usefulness and integration into existing workflows seemed to exist, which hindered stakeholder onboarding and optimal process flows in the notification cascade. Perceived lack of usefulness is also affecting the uptake of the DPT app in the population. However, “sense-making” by different participants may also be context- and time-dependent. The SwissCovid app was released at a time of relatively low SARS-CoV-2 incidence, which meant that DPT effects could not become apparent immediately [45]. However, in late August 2020, the FOPH presented data indicating rising numbers of app users who sought PCR testing after a SwissCovid notification and then tested positive, which may have alleviated some of the concerns regarding effectiveness.

It is worth noting that public agenda setting also played a role in media reports, as illustrated by articles including statements of DPT developers or reports covering FOPH press conferences. For example, in one article (#6, Multimedia Appendix 1), a member of the DPT development voiced concerns about insufficient engagement of some cantonal authorities in the DPT notification cascade. In another example (#37, Multimedia Appendix 1), the FOPH presented preliminary data from an effectiveness analysis to increase public confidence and ultimately app uptake in the population, which was followed by an increase in the number of active users around the end of August 2020 [43].

### Contribution to the Literature

The present analysis may contribute to the international debate on DPT on two levels. First, it provides insights into challenges of DPT implementation from a country with one of the longest track records of DPT implementation and a complex, federalistic health system. Second, by applying the NPT framework to classify different reports, the analysis also contributes on a methodological level by illustrating the usefulness of the NPT approach. NPT can provide guidance on how to bring complex digital health interventions to fruition by considering motivations, potential benefits, or operational burdens of a digital health intervention for different stakeholders who should engage in implementation. NPT postulates that individual and collective actions need to be in synchronization for a complex intervention to be successful. To quote one of the foundational papers of NPT: “The starting point of the theory is that to understand the embedding of a practice we must look at what people actually do and how they work [46].”

The present analysis indicates that NPT can indeed provide a useful framework to classify DPT challenges and may help to identify suitable optimization. For example, as suggested by the *reflexive monitoring* construct, some media reports indicate continuous DPT process adaptations, as well as constant communication with actors and assessments of potential improvements. Ideally, NPT or similar implementation frameworks should already be considered during the development and release of novel technologies, which was hindered in Switzerland by immense time pressures created by the pandemic situation.

### Study Limitations

Some limitations of the study and its interpretation are worth noting. The reliance on published media reports (and not, for example, on stakeholder interviews) may have limited the diversity and level of detail of the current debates about DPT. It may also have missed aspects that were never raised by the media but were discussed bilaterally between different actors. The reliance on published media reports was an advantage, as it enhanced the reproducibility of our study. Furthermore, it should be noted that the challenges outlined in this report do not reflect the status quo, and it is not intended to pass judgment on the success of DPT or the roles of the different participants in Switzerland. Finally, extraction and classification of the media reports was performed by a single person, which should also be considered as a limitation.

To summarize, the analysis of media reports on implementation challenges for DPT in Switzerland demonstrates that the nontechnical implementation of DPT must not be forgotten. The experiences in Switzerland indicate that the technical aspects work well, but in some instances, the nontechnical processes led to bottlenecks in the notification chain. This is understandable given the multiple interactions required between different participants. The lessons from Switzerland, one of the earliest adopters of DPT, and the demonstration of the usefulness of NPT for planning and analyzing NPT implementation will hopefully serve as an inspiration for other countries that are developing their own DPT implementations.

## Appendix

Multimedia Appendix 1 Articles that were included in the topic analysis.

## Abbreviations

API: application programming interface
DP-3T: Decentralized Privacy-Preserving Proximity Tracing
DPT: digital proximity tracing
FOPH: Federal Office of Public Health
MCT: manual contact tracing
NPT: normalization process theory
PCR: polymerase chain reaction
PEPP-PT: Pan-European Privacy-Preserving Proximity Tracing
TTIQ: test-trace-isolate-quarantine
