# Supplementary material for: Challenges for Nontechnical Implementation of Digital Proximity Tracing During the COVID-19 Pandemic: Media Analysis of the SwissCovid App
Source: JMIR Mhealth Uhealth. 2021 Feb 26;9(2):e25345. doi: 10.2196/25345 (PMC7919847; doi:10.2196/25345)
Supplement: Multimedia Appendix 1 [file mhealth_v9i2e25345_app1.docx]

Supplementary Table: Articles that were included in the topic analysis (n=38)^a^.

| **File #** | **Title** | **Date** | **Medium** |
| --- | --- | --- | --- |
| 1 | «Wir haben viel Grund zur Hoffnung» | 27.09.2020 | SonntagsZeitung |
| 2 | Corona-App erhält Daten oft zu spät | 14.08.2020 | NZZ |
| 3 | So ist die SwissCovid-App komplett nutzlos | 14.08.2020 | NZZ |
| 4 | Digitale Verwaltung braucht Transparenz | 23.07.2020 | NZZ |
| 5 | Geht der SwissCovid App die Luft aus? | 16.07.2020 | NZZ |
| 6 | «Es muss jemand auf den Tisch hauen» | 28.08.2020 | TagesAnzeiger |
| 7 | SP fordert dringend Verbesserungen beim Contact-Tracing | 28.08.2020 | Aargauer Zeitung |
| 8 | Kanton verschickt am Sonntag keine Covid-Codes | 27.08.2020 | Aargauer Zeitung |
| 10 | Epidemiologe kritisiert Behörden | 23.07.2020 | NZZ |
| 11 | Noch ein Rückschlag für die Corona-App | 21.07.2020 | Aargauer Zeitung |
| 12 | Siebzig Covid-Meldungen in sieben Tagen | 14.07.2020 | Aargauer Zeitung |
| 14 | Ärgerliche Corona-Test-Odyssee | 06.08.2020 | regio.ch |
| 15 | «Generelles Unbehagen» gegen Covid-App | 29.08.2020 | Bieler Tagblatt |
| 17 | Die Corona-App bleibt stumm | 20.08.2020 | St. Galler zeitung |
| 18 | Zu wenig Anrufer: Bund weiss noch nicht, ob Corona-App hilft | 16.07.2020 | blick.ch |
| 19 | Warum versagen die Kantone bei der SwissCovid-App? Es gibt einen bösen Verdacht | 28.08.2020 | watson.ch |
| 20 | Covid-App-Frust und ein wenig Hoffnung: So lief die Corona-Konferenzin Bern | 19.08.2020 | watson.ch |
| 22 | Das sind die grössten «Baustellen» bei der SwissCovid App | 07.07.2020 | watson.ch |
| 25 | «La Suisse n’était pas préparée à la bonne crise» | 28.07.2020 | le temps |
| 27 | Une application qui sauve des vies | 29.08.2020 | le temps |
| 28 | L’utilité de l’app SwissCovid se dessine | 29.08.2020 | le temps |
| 29 | Alarm ins Nirgendwo | 27.08.2020 | WOZ |
| 30 | Daniel Koch bot mehrmals seinen Rücktritt aus der Taskforce an | 12.09.2020 | TagesAnzeiger |
| 32 | Wie das BAG die SwissCovid-Nutzung ankurbeln will | 28.08.2020 | netzwoche.ch |
| 33 | SwissCovid: App-Entwickler Mathias Wellig über Tücken des Contact-Tracings | 27.08.2020 | netzwoche.ch |
| 35 | Corona-Fehlalarme bei Pflegepersonal | 05.09.2020 | Basler Zeitung |
| 36 | Schwarzpeterspiel um Corona Tracing | 29.08.2020 | Blick |
| 37 | Erstmals unerwartete Coronafälle entdeckt: "Wir haben den Sinn der App bewiesen" | 28.08.2020 | Aargauer Zeitung |
| 39 | Von wegen «ein Witz»: So reagieren die Kantone auf die harte Corona App-Kritik | 20.08.2020 | watson.ch |
| 42 | Schweizer vertrauen der Swiss-Covid App mehr als den Onlineshops | 21.08.2020 | 20 minuten online |
| 48 | Die Corona-App bringt mich um den Schlaf | 17.07.2020 | Basler Zeitung |
| 50 | Die Swiss-Covid-App alarmiert nicht immer | 26.07.2020 | Sonntagszeitung |
| 52 | Der Funke will nicht springen | 23.07.2020 | Weltwoche |
| 53 | Sandro Brotz motzt gegen Swiss-Covid-App | 22.07.2020 | blick.ch |
| 57 | Gewarnte gehen nicht über Infoline | 17.07.2020 | TagesAnzeiger |
| 58 | Wirkung der Swiss-Covid-App kann noch nicht genau gemessenwerden | 16.07.2020 | Basler Zeitung |
| 60 | Ringen um Akzeptanz und Wirksamkeit | 11.07.2020 | Schafhauser Nachrichten |
| 61 | Angst vor zu vielen Fehlalarmen | 09.07.2020 | Linth-Zeitung |

^a^Links to the articles cannot be provided because they are either print articles or are behind paywalls. PDF files are available from the author upon reasonable request.
